# Supplementary material for: Auditory cortex conveys non-topographic sound localization signals to visual cortex
Source: Nat Commun. 2024 Apr 10;15:3116. doi: 10.1038/s41467-024-47546-4 (PMC11006897; doi:10.1038/s41467-024-47546-4)
Supplement: Supplementary file 1 — Supplementary Information [file 41467_2024_47546_MOESM1_ESM.pdf]

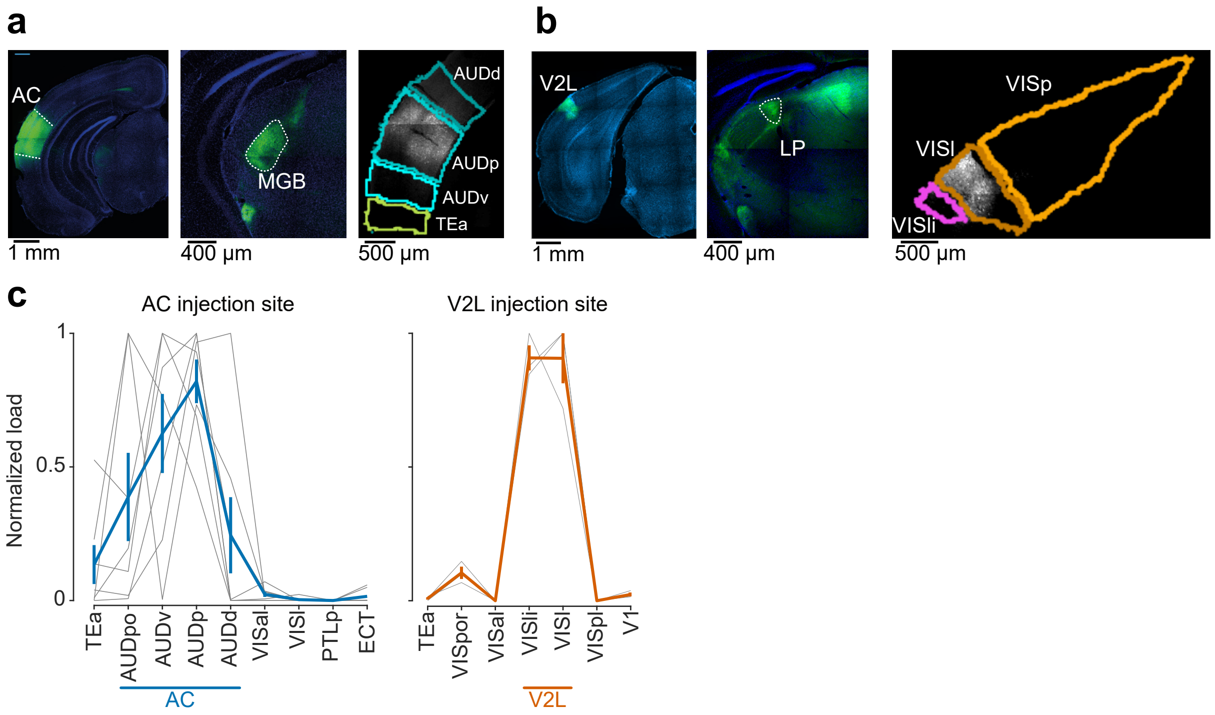

Supplementary Figure 1. Histological analysis of AC- and V2L- injected mice. a, Example coronal sections of an AC-injected mouse. Left, Section through the injection site in the AC cortex. Center, axonal projections in the auditory thalamus. Right, sections were registered to the Allen mouse brain atlas to estimate the relative GCaMP expression across the AC and neighboring regions. **b,** Same for an example V2L-injected mouse. **c,** Quantification of fluorescence in different areas around the injection site for AC- (blue, left) and V2L-injected mice (yellow, right). Gray lines, individual mice; colored line, mean ± s.e.m. across mice. n = 7 AC-injected mice and n = 3 V2L-injected mice. Auditory areas: AC, auditory cortex; AUDpo, posterior auditory cortex; AUDd, dorsal auditory cortex; AUDp, primary auditory cortex; AUDv, ventral auditory cortex; MGB, medial geniculate body; Visual areas: V2L, higher-order lateral visual area; V1, primary visual cortex; VISpor, postrhinal area; VISal, anterolateral visual area; VISl, lateral visual area; VISli, laterointermediate visual area; VISpl, posterolateral visual area; LP, lateroposterior nucleus; Others: TEa, temporal association areas; PTLp, posterior parietal association area; ECT, ectorhinal area.

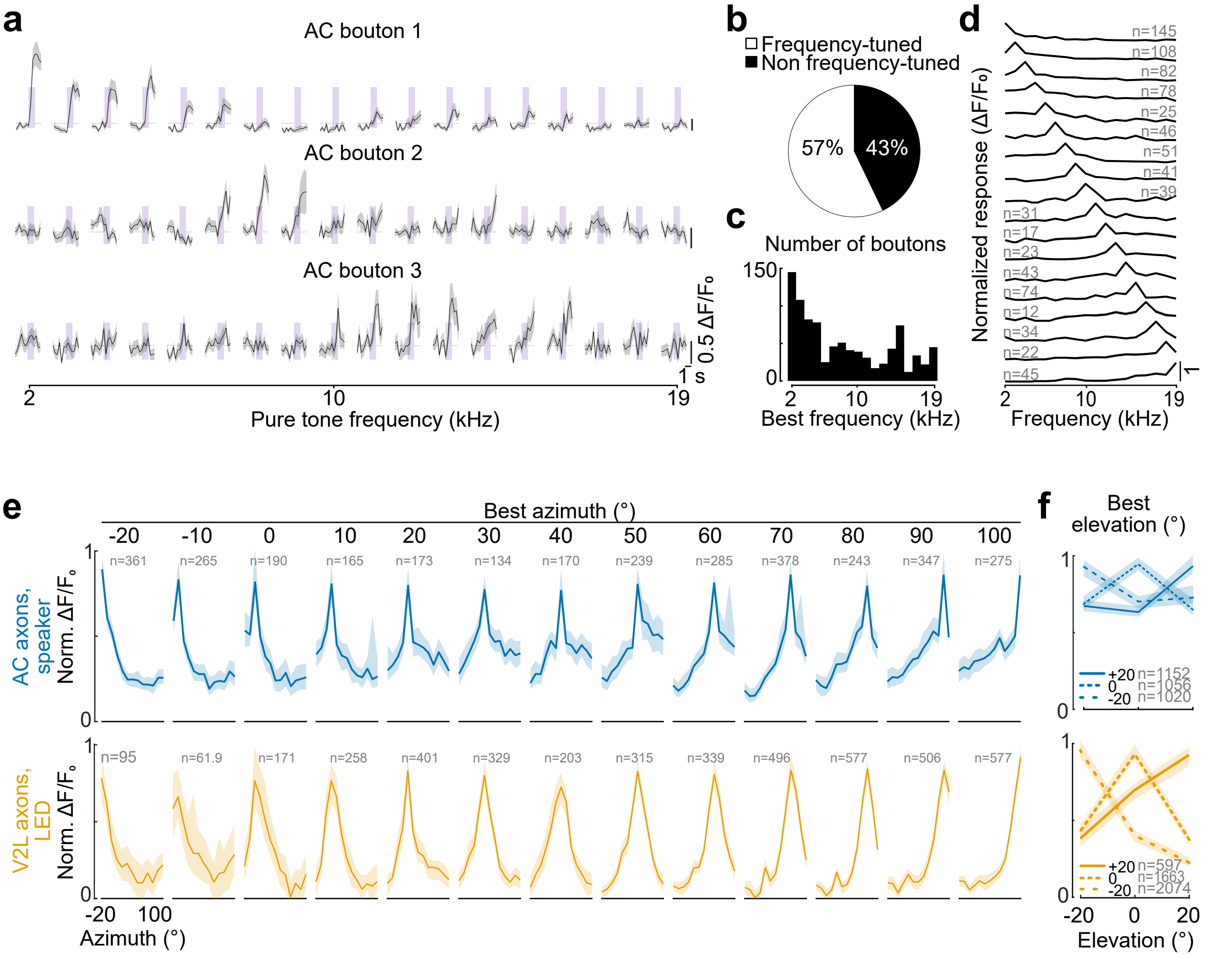

Supplementary Figure 2. Frequency and spatial tuning in AC→V1 inputs. a, Average responses to different pure tone frequencies (2-19 kHz, 1 kHz step, purple shaded area) from three example AC boutons recorded in the same imaging session. **b,** Fraction of responsive boutons that are frequency-tuned (one-way repeated measure ANOVA, $p < 0.05$). n = 916 boutons from 5 mice. **c,** Distribution of best frequencies. **d,** Normalized frequency tuning curves. Each plot is the mean of all the boutons with the same best frequency. **e,** Normalized azimuth tuning curves. Each plot is the mean of all the cross-validated boutons preferring sounds located at a given azimuthal position. Colored lines, mean across boutons; shaded area, 95% confidence interval. The mean number of boutons across resamplings is indicated in gray. **f,** Same as **e** for elevation.

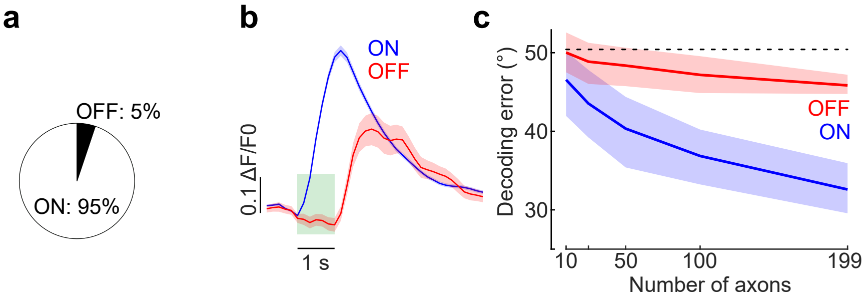

Supplementary Figure 3. Onset responsive boutons dominates in AC→V1 inputs and convey spatial information. a, Proportions of sound onset (ON) vs. offset (OFF) responsive boutons (n = 4820 vs 262, respectively). **b,** Average response to best speaker position across all ON vs. OFF boutons. Data is mean ± s.e.m. across boutons; green shaded area, auditory stimulus presentation. **c,** Decoding error as a function of number of axons for ON vs. OFF axons. Curve and shaded area, mean and 95% confidence interval across 100 resampling iterations; dashed line, chance.

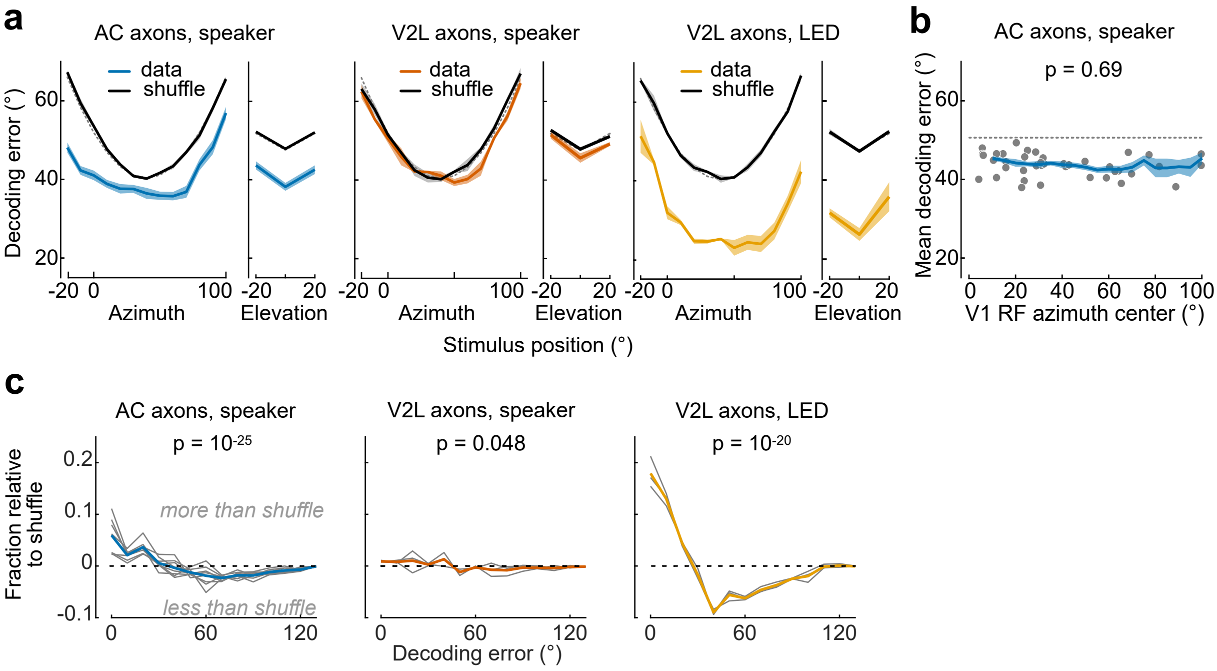

Supplementary Figure 4. Decoding of stimulus location from AC and V2L inputs in V1. **a,** Decoding error as a function of stimulus azimuthal (left) and elevation location (right) in auditory responses in AC axons (left) and V2L axons (middle) and in visual responses in V2L axons (right). Colored lines, average across mice; black lines, shuffled data. Shaded areas are the s.e.m. across mice. n = 8 AC- and n = 3 V2L-injected mice. Dashed lines are chance level. **b,** Mean decoding error as a function of imaging position in V1, using matched number of axons per position (23 axons per positions, n = 41 positions). V1 RF azimuth center measured using LED responses in jRGECO1a-expressing somatas in L2/3 of V1. Dots, individual sessions; curve and shaded area, moving average and s.e.m calculated over 10° bins. One-way ANOVA. **c,** Distribution of decoding errors, subtracted by that of the shuffle data. Thin gray lines, individual mice; thick lines, average across. One-way repeated measures ANOVA.

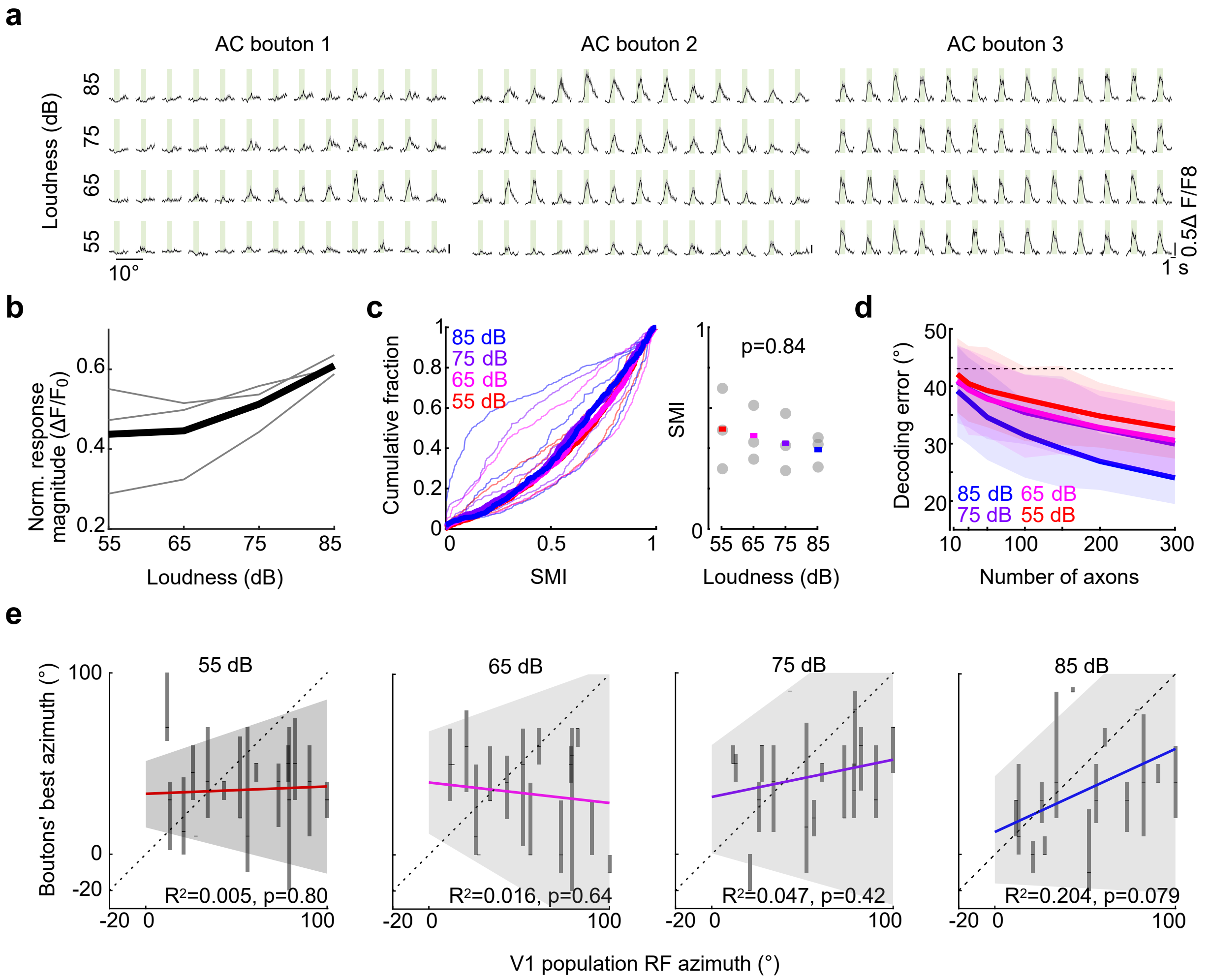

Supplementary Figure 5. Spatial tuning at various loudness levels. a, Example AC bouton responses to sound across different azimuthal positions and loudness levels. **b,** Magnitude of the auditory responses averaged across speaker positions, normalized by the maximum response across loudness levels. All the boutons responsive to any of the loudness are plotted. Gray lines, individual mice; black line, average across mice. n = 3 mice, 5189 boutons. **c,** SMI vs loudness. Only boutons responsive at each loudness are plotted (1486, 1856, 2370 and 2920 boutons from 3 mice for 55, 65, 75 and 85 dB, respectively). Left, cumulative SMI distribution. Thin lines, individual mice, color-coded for loudness; thick lines, cumulative distribution across mice. Anderson-Darling 4-sample test, $p = 8x10^{-12}$. Right, mean SMI across mice. Circles, mice; tick, average across mice. One-way repeated measure ANOVA, n = 3 mice. **d,** Decoding error as a function of number of axons for different loudness levels. Data is mean and 95% confidence interval across 100 resampling rounds; dashed line, chance level. **e,** Mean best azimuth of the sound-evoked response across AC boutons as a function of the population RF center of V1 neurons for each imaging session. Left to right, increasing loudness. n = 16 imaging sessions, 3 mice. Black ticks and gray shading, median and 95% confidence interval. Colored lines and values correspond to the linear regression of the mean values; gray shading, 95% confidence interval. Dashed lines, identity lines

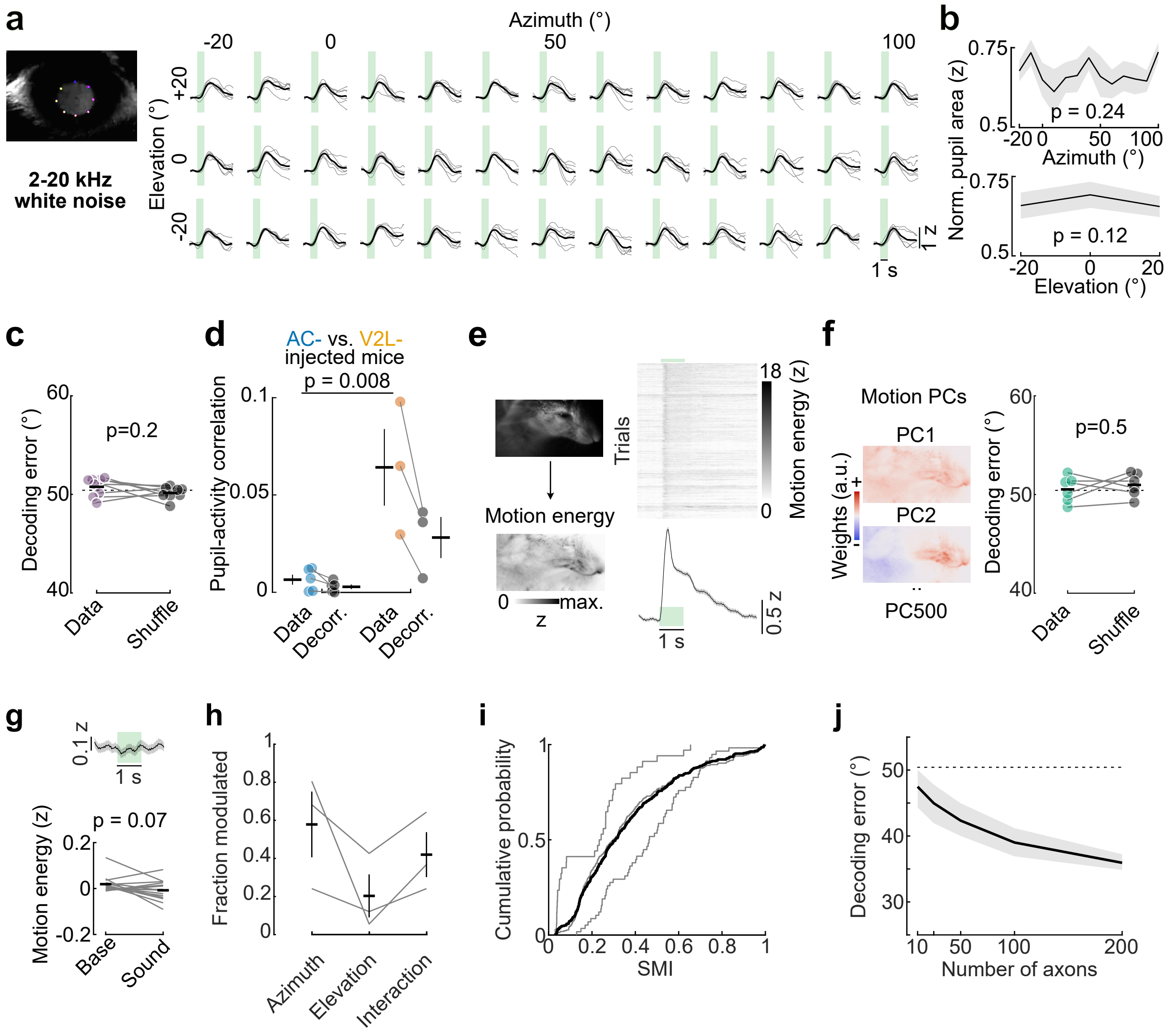

Suplementary Figure 6. Sound-induced behavioral responses are similar across space. a-d, Pupil response to 2-20 kHz, 85 dB white noise. **a,** Left, Example video frame showing the pupil contour tracked by Deeplabcut. Right, Sound-induced pupil responses for sounds played at different positions of the speaker array for an example mouse. Green shaded area, auditory white noise; gray lines, sessions; black lines, average across sessions. **b,** Pupil responses across azimuthal (left) and elevation (right) showed no systemic bias (one-way repeated measure ANOVA, n = 8 mice: 5 AC- and 3 V2L-injected mice). Data is mean ± s.e.m. across mice. **c,** Sound localization decoding error using pupil area (purple) versus the trial shuffle data (black). Circles, individual mice; black ticks, average; dashed line, chance. Two-sided paired t-test, n = 8 mice. **d,** Pearson's correlation between pupil area and auditory responses in AC (blue) and V2L boutons (yellow). Circle, average correlation across boutons per mice. Crosses, mean ± s.e.m; gray, corresponding trial-shuffle data ('decorrelated'). Pupil-bouton pairwise correlations were larger in V2L- than in AC-injected mice (two-sided t-test). In AC-injected mice, pairwise correlations were not significantly different between actual and decorrelated data (two-sided paired t-test: p = 0.09). **e-f,** Facial response to 2-80 kHz, 65 dB white noise in all sessions of CBA mice. **e,** Example video motion energy from one session. Left, average across all frames (top) and average motion energy (bottom). Right, motion energy for all trials (top) and averaged across trials (bottom). **f,** Left, spatial masks of the top two principal components (PC) of the motion energy obtained from the example in **e**. Color scale indicates low- (blue) to high-motion energy (red). PC1 captures motion anywhere on the mouse's face while PC2 highlights movement in the snout area. Right, sound location decoding error from the 500 first PCs of the face movie (green) was not significantly higher than that of the trial-shuffle data (gray; two-sided paired t-test, n = 6 CBA mice). Dots, individual mouse, data averaged across session; black tick, group average; dotted line, chance level. **g-j,** Facial response in sessions where 2-80 kHz, 65 dB white noise did not drive significant behavioral enhancement (15 sessions from 2 CBA and 1 Thy1-jRGECO1a mouse). **g,** Top, example session motion energy trace, averaged across all trials. Same scales as in **e.** Bottom, quantification across all sessions (two-sided paired t-test). **h,** Fraction of boutons significantly modulated by speaker position ($p < 0.05$, two-way ANOVA). Gray lines, individual mice; Cross, mean ± s.e.m. across mice. **i,** Cumulative SMI distribution. Gray lines, individual mice; black line, distribution from all mice. **j,** Decoding error as a function of number of axon used. Black and shaded area are mean and 95% confidence interval. Dashed line, chance.

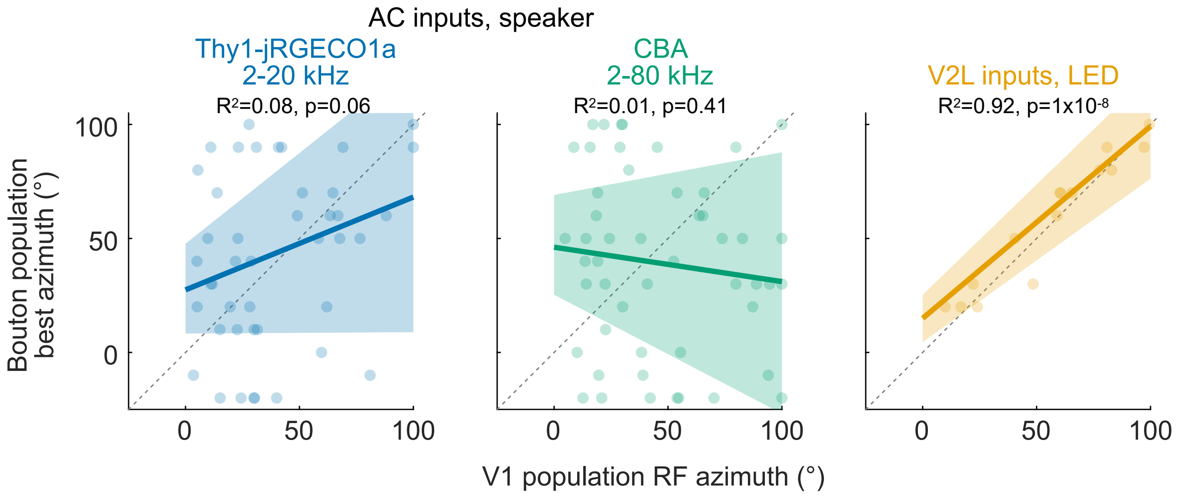

Supplementary Figure 7. Lack of topographical organization of the mean AC bouton population response to sounds. Best azimuth of the mean bouton population response as a function of the population RF azimuth in V1 neurons. Similar analysis as in Figure 4b but responses across all boutons were first averaged and then the best azimuth of then mean was calculated. Thy1-jRGECO1a mice, 2-20 kHz: n = 43 imaging sessions; CBA mice, 2-80 kHz: n = 50 imaging sessions; V2L inputs: n = 15 imaging sessions. Circles, individual imaging session; solid lines, linear fit; shaded area, 95% confidence interval. Dashed line, identity line.

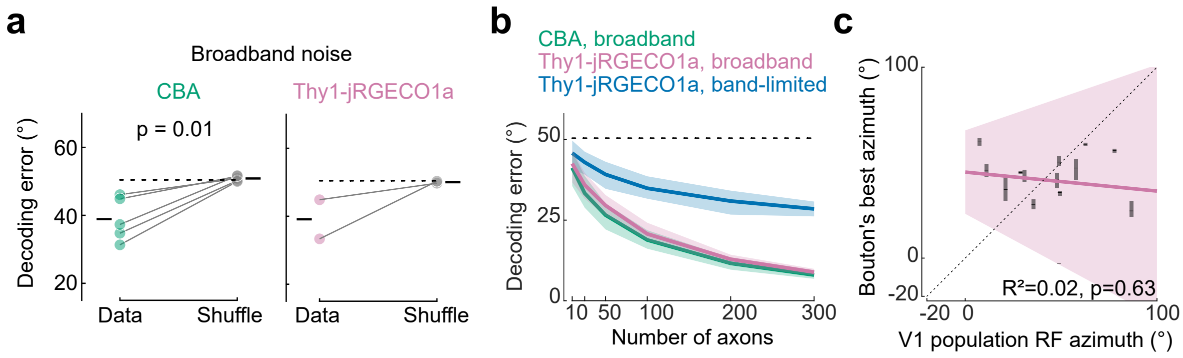

Supplementary Figure 8. Including high-frequencies improves decoding of sound location from AC→V1 inputs but did not reveal a topographic organization. a, Sound location decoding error using high-frequency containing sound (2-80 kHz white noise) in CBA (left) and Thy1-jRGECO1a mice (right). Colored circles, average across sessions for each mouse; gray circles, same for shuffle data; black tick, average across mice; dashed lines, chance level. Two-sided paired t-test: n = 5 CBA mice, 30 sessions and 2 Thy1-jRGECO1a mice, 14 sessions. **b,** Sound location decoding error as a function of number of axons for Thy1-jRGECO1a mice, band-limited noise (blue), broadband noise (purple) and CBA mice, broadband noise (green-blue). Lines and shaded areas are means and 95% confidence intervals; dashed line, chance. **c,** Mean peak azimuth of the sound evoked response across AC boutons as a function of the population RF center of V1 neurons for each imaging session. Ticks, median; gray shading, 95% confidence interval. Colored lines, linear fits of the median values; colored shading, 95% confidence interval; dashed line, identity line. n = 14 sessions, 2 mice.

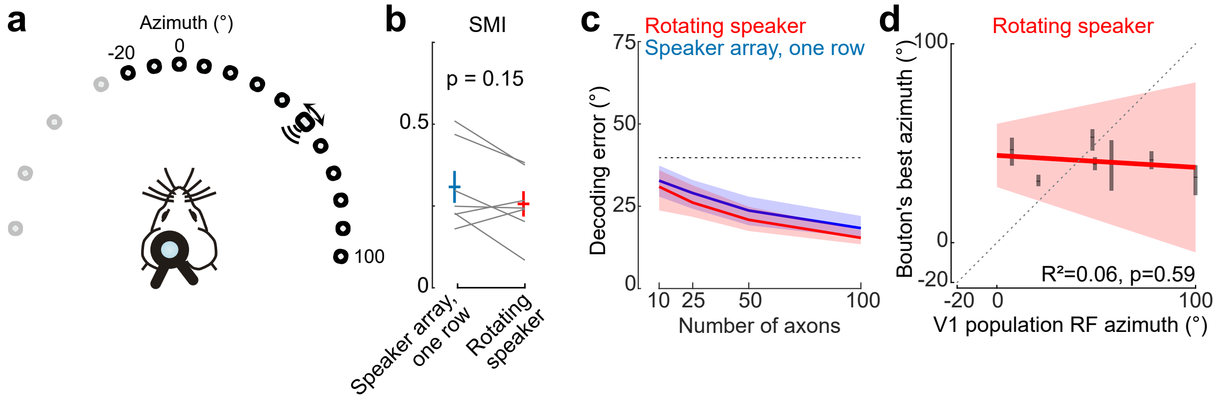

Supplementary Figure 9. Sound location specific signals in AC inputs to V1 measured using a single loudspeaker. a, A single loudspeaker (black rectangle, broadband white noise) was moved between -20° to +100° in azimuth and 0° elevation, 10° spacing (black rectangles). Light gray positions represent positions sampled during the same experiment, analyzed separately (see **Figure 5**). **b,** Similar SMI values were obtained with a rotating speaker and with the middle row (0° elevation) of the speaker and LED array (two-sided paired t-test, n = 7 imaging sessions from 2 mice). **c,** Sound location decoding error as a function of number of axons used was similar across both conditions. Lines and shaded area are means and 95% confidence intervals; dashed line, chance level. **d,** AC bouton's best azimuth are not topographically organized according to V1 retinotopy (n = 7 sessions from 2 mice).

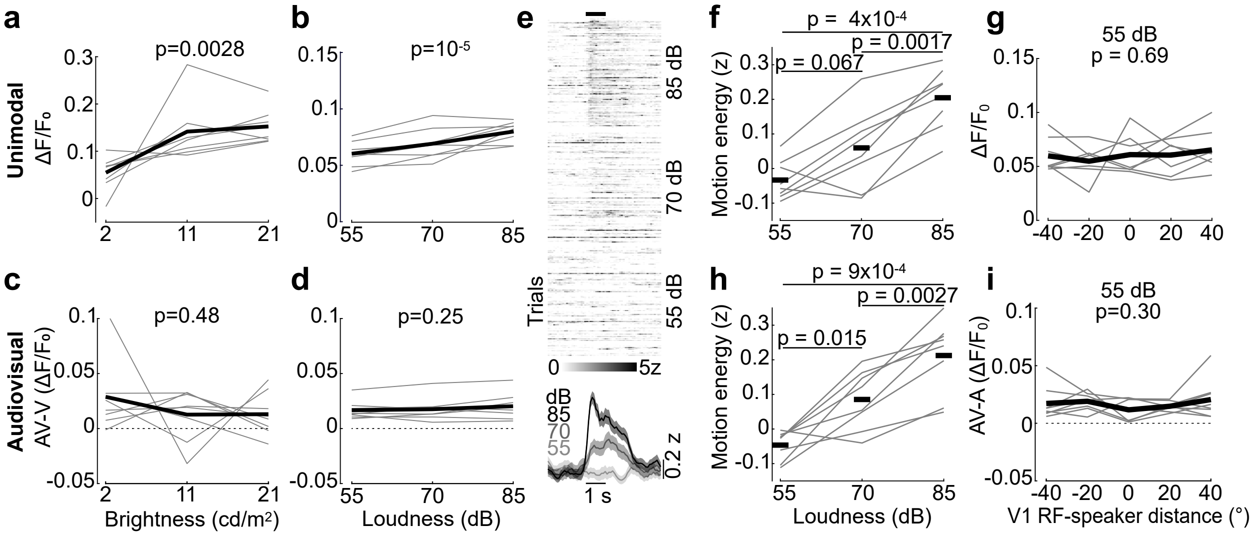

Supplementary Figure 10. Characterization of audiovisual responses across stimulus intensities and in sessions without discernible sound-induced behavioral responses. a-b, Magnitude of visual (**a**) and auditory (**b**) responses as a function of stimulus intensity. One-way repeated-measure ANOVA, n = 8 mice. **c-d,** Magnitude of AV responses as a function of brightness (averaged across brightness levels in **c**) or loudness (averaged across brightness levels, in **d**). **e,** Face motion energy across trials for an example session, sorted by loudness (auditory and AV trials). Horizontal bar, sound presentation. Bottom, average per loudness. **f,** Face motion energy upon auditory-only stimulation across the three different loudness levels. Tukey's post-hoc test after one way repeated-measure ANOVA ($p = 10^{-6}$), n = 8 mice. **g,** Neuronal response magnitude to auditory stimulation at a loudness with no discernible sound-induced facial motion energy enhancement (55 dB). One-way repeated-measure ANOVA, n = 8 mice. **h-i,** same as for **f-g** for AV trials. One-way repeated-measure ANOVA, $p = 10^{-6}$ (**h**), n = 8 mice. AV responses where averaged across brightness levels.

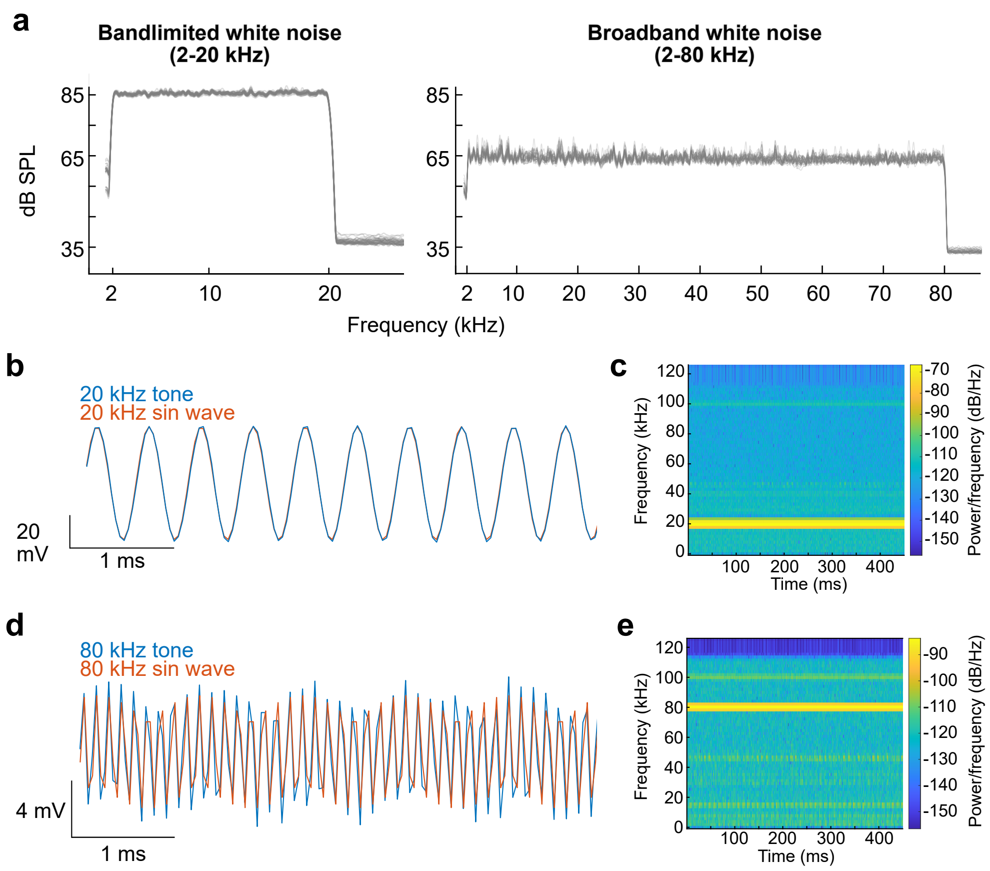

Supplementary Figure 11. Speaker calibration and single tone responses. a, Power spectrum of the bandlimited and broadband white noises for the 39 speakers (gray) after an example round of equalization. **b,** Recorded output to a 20 kHz tone (blue) vs. sin wave generated at the same sample frequency (red, 192 kHz). **c** Spectrogram of the recorded 20 kHz tone. **d,e,** same for a 80 kHz tone.

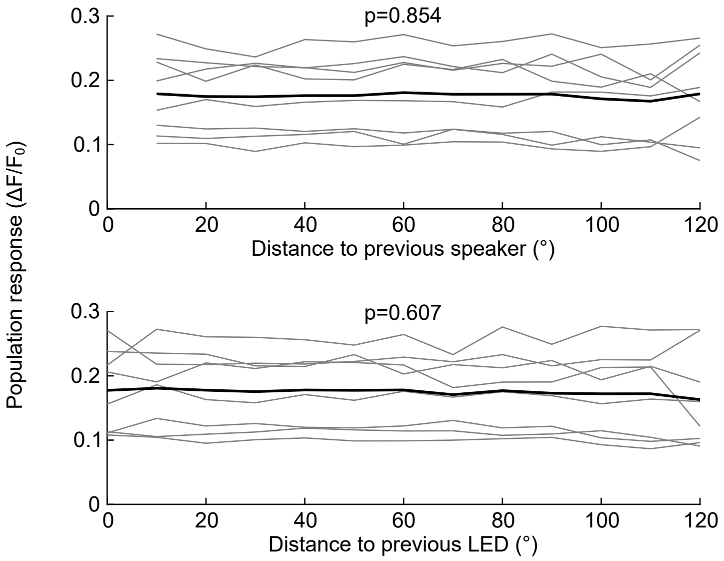

Supplementary Figure 12. The population response of AC boutons in V1 does not depend on the distance between current speaker position and that of the previous stimulus. Gray, individual mice; black, average across mice; One-way repeated measure ANOVA: distance to previous speaker, $F(11,77) = 0.56$, distance to previous LED, $F(11,77) = 0.61$; $n = 8$ mice.
