# Supplementary material for: Auditory cortex conveys non-topographic sound localization signals to visual cortex
Source: Nat Commun. 2024 Apr 10;15:3116. doi: 10.1038/s41467-024-47546-4 (PMC11006897; doi:10.1038/s41467-024-47546-4)
Supplement: Supplementary file 3 — Reporting Summary [file 41467_2024_47546_MOESM3_ESM.pdf]

nature portfolio

Corresponding author(s): Leopoldo Petreanu
Camille Mazo

Last updated by author(s): Dec 5, 2023

Reporting Summary

Nature Portfolio wishes to improve the reproducibility of the work that we publish. This form provides structure for consistency and transparency in reporting. For further information on Nature Portfolio policies, see our Editorial Policies and the Editorial Policy Checklist.

Statistics

For all statistical analyses, confirm that the following items are present in the figure legend, table legend, main text, or Methods section.

n/a	Confirmed	
☐	☒	The exact sample size (*n*) for each experimental group/condition, given as a discrete number and unit of measurement
☐	☒	A statement on whether measurements were taken from distinct samples or whether the same sample was measured repeatedly
☐	☒	The statistical test(s) used AND whether they are one- or two-sided<br>*Only common tests should be described solely by name; describe more complex techniques in the Methods section.*
☒	☐	A description of all covariates tested
☐	☒	A description of any assumptions or corrections, such as tests of normality and adjustment for multiple comparisons
☐	☒	A full description of the statistical parameters including central tendency (e.g. means) or other basic estimates (e.g. regression coefficient) AND variation (e.g. standard deviation) or associated estimates of uncertainty (e.g. confidence intervals)
☐	☒	For null hypothesis testing, the test statistic (e.g. *F*, *t*, *r*) with confidence intervals, effect sizes, degrees of freedom and *P* value noted<br>*Give P values as exact values whenever suitable.*
☒	☐	For Bayesian analysis, information on the choice of priors and Markov chain Monte Carlo settings
☒	☐	For hierarchical and complex designs, identification of the appropriate level for tests and full reporting of outcomes
☐	☒	Estimates of effect sizes (e.g. Cohen's *d*, Pearson's *r*), indicating how they were calculated

Our web collection on statistics for biologists contains articles on many of the points above.

Software and code

Policy information about availability of computer code

Data collection	ScanImage (Vidrio Technologies; https://vidriotechnologies.com/),<br>Zeiss AxioImager 2<br>Bonsai
Data analysis	Matlab2020a (data and statistical analysis)<br>Suite2p (https://github.com/MouseLand/suite2p)<br>Facemap (https://github.com/MouseLand/facemap)<br>Deeplabcut(https://github.com/DeepLabCut/DeepLabCut)<br>Fiji<br>Matlab Custom code (https://github.com/camille-lab/ACaxonsSpace/)

For manuscripts utilizing custom algorithms or software that are central to the research but not yet described in published literature, software must be made available to editors and reviewers. We strongly encourage code deposition in a community repository (e.g. GitHub). See the Nature Portfolio guidelines for submitting code & software for further information.

Data

Policy information about availability of data

All manuscripts must include a data availability statement. This statement should provide the following information, where applicable:

- Accession codes, unique identifiers, or web links for publicly available datasets
- A description of any restrictions on data availability
- For clinical datasets or third party data, please ensure that the statement adheres to our policy

The data supporting the main figures is deposited in Zenodo (https://doi.org/10.5281/zenodo.10685211)

Research involving human participants, their data, or biological material

Policy information about studies with human participants or human data. See also policy information about sex, gender (identity/presentation), and sexual orientation and race, ethnicity and racism.

Reporting on sex and gender	N/A
Reporting on race, ethnicity, or other socially relevant groupings	N/A
Population characteristics	N/A
Recruitment	N/A
Ethics oversight	N/A

Note that full information on the approval of the study protocol must also be provided in the manuscript.

Field-specific reporting

Please select the one below that is the best fit for your research. If you are not sure, read the appropriate sections before making your selection.

☒ Life sciences ☐ Behavioural & social sciences ☐ Ecological, evolutionary & environmental sciences

For a reference copy of the document with all sections, see nature.com/documents/nr-reporting-summary-flat.pdf

Life sciences study design

All studies must disclose on these points even when the disclosure is negative.

Sample size	Sample size was estimated based on the expected effect size based on similar studies (Marques et al 2018, Iurilli et al 2012) and the current standard in mouse neuroscience studies .
Data exclusions	Two-photon recordings with poor signal/data quality were excluded as assessed by visual inspection of the registered time-series images and registration metrics. Poor data quality usually originates from movement artifacts during axon imaging of awake mice. Data exclusion was done prior to analysis, in order not to bias exclusion criteria. Neurons and boutons with low signal to noise ratio during sensory stimulation were excluded from analysis as specified in Methods. While we confirmed the accuracy of the AAV injections in targeting the intended brain areas through histological analyses, no mice with recordings of good signal quality were excluded based on these analyses.
Replication	Experiments were performed independently in different mice and statistics were performed across mice in most cases.<br>The observation of the presence of spatial-confined RFs in AC inputs and the lack of topographical organization in V1 was successfully replicated in 3 independent groups of mice (two groups of C57BL/6 and one of CBA mice) using the speaker array. They were also succesfully replicated using a single speaker, rotated around the head in C57BL/6 mice.
Randomization	Stimulus presentation was randomized as described in the Methods. Mice were randomly assigned to the different experimental groups.
Blinding	Obvious differences to the expert eye in the morphological features of axons from AC- and V2L-injected mice (or C57BL/6 and CBA mice) did not allow for blind data collection and analysis.<br>However, data from AC- and V2L-injected (C57BL/6 and CBA mice) mice was collected using the same experimental design and data analysis was performed using the same pipeline and criteria. Consequenlty the measurements are not affected by the lack if blinding.

Reporting for specific materials, systems and methods

We require information from authors about some types of materials, experimental systems and methods used in many studies. Here, indicate whether each material, system or method listed is relevant to your study. If you are not sure if a list item applies to your research, read the appropriate section before selecting a response.

Materials & experimental systems

n/a	Involved in the study	
☐	☒	Antibodies
☒	☐	Eukaryotic cell lines
☒	☐	Palaeontology and archaeology
☐	☒	Animals and other organisms
☒	☐	Clinical data
☒	☐	Dual use research of concern
☒	☐	Plants

Methods

n/a	Involved in the study	
☒	☐	ChIP-seq
☒	☐	Flow cytometry
☒	☐	MRI-based neuroimaging

Antibodies

Antibodies used

polyclonal anti-GFP antibody, dilution 1:4000; ThermoFisher, catalog #A-6455
Alexa Fluor 488-conjugated secondary antibody, dilution 1:1000; ThermoFisher, catalog #A-11008

Validation

These antiobodies have been extensively used for immunohistochemistry purposes by the scientific community with numerous species-relevant citations on the manufacturer website.
Polyclonal anti-GFP antibody: https://www.thermofisher.com/antibody/product/GFP-Antibody-Polyclonal/A-6455
Alexa Fluor 488-conjugated secondary antibody: https://www.thermofisher.com/antibody/product/Goat-anti-Rabbit-IgG-H-L-Cross-Adsorbed-Secondary-Antibody-Polyclonal/A-11008

Animals and other research organisms

Policy information about studies involving animals; ARRIVE guidelines recommended for reporting animal research, and Sex and Gender in Research

Laboratory animals

Animals used in this study were of the species Mus musculus and the strains: C57BL/6, breed-in house, Thy1-jRGECO1a (Tg(Thy1-jRGECO1a)GP8.20Dkim/J, MGI:J:268005, JAX stock #030525), Ai148D (Ai148(TIT2L-GC6f-ICL-tTA2)-D, JAX stock #030328) crossed with Slc17a7-IRES2-Cre (or Vglut1-IRES2-Cre-D, JAX stock #023527), and CBA/CaCrl (Charles River Laboratories, stock #609). For mice of all the strains, adult (8-9 weks old) male and female mice were used in this study.

Wild animals

The study did not involve wild animals

Reporting on sex

Data was collected from 16 males and 8 females. Th1-jRGECO1a and Ai148DxSlc17a7-IRES-Cre mice were bred in-house and experiments were performed in both males or females, depending on availability (Th1-jRGECO1a: 6 males and 5 females; Ai148DxSlc17a7-IRES-Cre: 2 males and 3 females). CBA mice were obtained from an external furnisher and were all males (n=8). Sex-based analyses were not performed due to otherwise too small sample size.

Field-collected samples

The study did not involve samples collected from the field

Ethics oversight

All animal procedures were reviewed by the Champalimaud Centre for the Unknown Ethics Committee guidelines and and approved by the Portuguese Veterinary General Direction (Ref.No.0421/000/000/2019)

Note that full information on the approval of the study protocol must also be provided in the manuscript.
